# Unmasking The Self – A Case of Body Dysmorphic Disorder

**DOI:** 10.1192/j.eurpsy.2026.11600

**Published:** 2026-07-31

**Authors:** F. G. Sousa, R. Simões, D. Mota, C. G. Pereira

**Affiliations:** Centro de Responsabilidade Integrado de Psiquiatria da Unidade Local de Saúde de Coimbra, Coimbra, Portugal

## Abstract

**Introduction:**

Body dysmorphic disorder (BDD) stands at the intersection of psychiatry and the silent language of the body, where the mirror reflects not reality but an unrelenting distortion. Patients often perceive themselves as disfigured or abnormal and sometimes have perceptions that brush into quasi-persecutory experiences, blurring the boundary with psychotic disorders. This sense of disfiguration can become magnified into an intolerable flaw, challenging clinicians to distinguish structural reality from psychopathological amplification.

**Objectives:**

Describe the case of a patient with an overwhelming preoccupation with mandibular retrognathism, who resorted to wearing a mask to hide what he perceived as unbearable deformity. Moreover, explore the diagnostic limbo between BDD and potential primary psychotic disorder.

**Methods:**

Comprehensive psychiatric assessment, including family interviews and detailed mental state evaluation, with particular attention to the patient’s persecutory-like ideation—specifically, his conviction that others were staring at or commenting on his mandibular appearance. Diagnostic formulation carefully weighed BDD against the possibility of psychosis-spectrum pathology.

**Results:**

Initially, the patient arrived persistently wearing a mask, symbolizing self-protection and self-image issues. He exhibited depressed mood, anhedonia, restricted affect, and rigid attribution of blame toward his mother for his perceived mandibular retrognathism. Psychotic phenomena, including persecutory ideation and possibly auditory-verbal due to inappropriate head turns and facial expressions, could not be excluded. Family engagement illuminated premorbid personality traits, mannerisms and the evolution of the symptoms.

**Conclusions:**

This case underscores the fragile threshold between BDD and psychotic-spectrum pathology, where distorted reflections of self manifest in both behavior and interpersonal relationships. Recognising such overlap is vital, as treatment relies on a multimodal intervention, integrating psychotherapy, pharmacotherapy, and family support are essential. The mask, rather than offering relief, symbolised the split between external concealment and internal torment.

**Disclosure of Interest:**

None Declared

